# Model explanation of the seasonal variation of δ^18^O in cow (*Bos taurus*) hair under temperate conditions

**DOI:** 10.1038/s41598-017-00361-y

**Published:** 2017-03-23

**Authors:** Guo Chen, Hans Schnyder, Karl Auerswald

**Affiliations:** Lehrstuhl für Grünlandlehre, Technische Universität München, Alte Akademie 12, Freising-Weihenstephan, 85354 Germany

## Abstract

Oxygen isotopes (δ^18^O) in animal and human tissues are expected to be good recorders of geographical origin and migration histories. However, seasonal variation of δ^18^O may diminish the origin information in the tissues. Here the seasonality of δ^18^O in tail hair was investigated in a domestic suckler cow (*Bos taurus*) that underwent different ambient conditions, physiological states, keeping and feeding during five years. A detailed mechanistic model was built to explain this variation. The measured δ^18^O in hair significantly related (p < 0.05) to the δ^18^O in meteoric water in a regression analysis. Modelling suggested that this relation was only partly derived from the direct influence of feed moisture. Ambient conditions (temperature, moisture) also affected the animal itself (drinking water demand, transcutaneous vapor etc.). The clear temporal variation thus resulted from complex interactions with multiple influences. The twofold influence of ambient conditions via the feed and via the animal itself is advantageous for tracing the geographic origin because δ^18^O is then less influenced by variations in moisture uptake; however, it is unfavorable for indicating the production system, e.g. to distinguish between milk produced from fresh grass or from silage. The model is versatile but needs testing under a wider range of conditions.

## Introduction

The potential use of the ^18/16^O isotope ratio values (δ^18^O) in animal tissues such as nail, hair, bone and feather to track geographic origin, climate and migration has been recognized during recent decades^1, 2^. It is based on empirical correlations between δ^18^O in animal tissues and the δ^18^O of amount-weighted mean annual (or mean-growing season) precipitation^3^. These correlations have been used to provide authentication of meat and dairy products^4–6^ and as tools for use in anthropology and archeology^7^. Among the different types of animal tissues, hair is preferred as the archival record of the animal’s diet because it grows continuously and preserves its isotopic information once formed^8^. Significant relations between the δ^18^O of tissue and that of precipitation have been found primarily in inorganic molecules such as phosphates and carbonates of bones and tooth enamel; however, an isotopic relation between hair and that of precipitation has not been found for all species (such as felids^3^). Although a general relation between δ^18^O in hair and that in precipitation was found for humans^9, 10^, it is still unknown whether δ^18^O in the hair of domestic animals such as cows can be used to track their geographic origin effectively, because their digestive systems differ from that of humans and their feed is also controlled by humans.

The challenge of interpreting δ^18^O in hair of domestic animals stems from several reasons. Unlike nitrogen and carbon, O has multiple input fluxes (air O, inhaled air water vapor, chemically bound O in feed, feed moisture and drinking water) and output fluxes (CO_2_ production, fecal water, milk water, exhaled water vapor, sweat water, transcutaneous water vapor, urea or uric acid and urine water) that are largely controlled by animal physiology and by environmental influences other than isotopic composition of meteoric water. Thus, the variation of δ^18^O in hair cannot directly reflect the variation of δ^18^O in precipitation. For instance, drinking water amount can be influenced by precipitation, temperature, relative humidity and plant available water, and also by intake of feed dry matter, crude protein and Na^11–15^, thereby causing a varying contribution of drinking water to total O input flux. The δ^18^O of water in grass, which is the main feed of cattle at pasture, changes hourly with ambient conditions^16^. Even δ^18^O of silage water varies with the fluctuation of ambient conditions and the change of exposure time^17^. Furthermore, the intercepted rain and dew in the grass can be ingested by grazing animals, which further increases the complexity of the O input of animals^15, 18^. This suggests that the δ^18^O of daily total feed moisture is a complex variable influenced not only by the ambient conditions but also by feeding time and frequency. The husbandry of domestic animals has additional influences on their diets and water intake. The feeding strategy changes with the season: in temperate latitudes fresh grass is usually fed in the warm season, which is the main growing period, whereas silage and hay are provided in winter; these sources differ in δ^18^O for both organic O and water O. Moreover, husbandry and feeding strategy will differ with production type and intensity (e.g., dairy vs beef; different milk yields among dairy systems). Finally, δ^18^O in hair is thought to be derived from exchange of amino-O with gut water^9^. Cows have a four-compartment stomach involved in water absorption and remixing; this makes the prediction of gut water more complicated than for monogastric animals. For these reasons, there is a need for a mechanistic, isotope-enabled δ^18^O model related to ambient conditions, and animal husbandry, including feeding and animal physiology, to examine the influence of different parameters on the seasonality of δ^18^O in hair.

Several models have described the δ^18^O of body water based on the isotopic balance of O^19–23^. However, these models did not link ambient conditions (such as temperature and humidity) and body water together, and they did not consider the influence of ambient conditions on the amount of O input fluxes. Kohn^2^ developed a general model to describe the relationships between δ^18^O of body water and the δ^18^O input fluxes (air O uptake, air water vapor into the lungs, chemically bound O in feed, feed moisture and drinking water) and output fluxes (CO_2_ production, fecal water, respiratory water, sweat water, transcutaneous water vapor, urea and urine) based on the isotopic balance and amount balance of O and the influence of ambient conditions. This model was used to analyze the sensitivity of climatic variation and species-specific differences in physiology on δ^18^O of body water and tissue O of different genera. The model successfully explained the phenomenon that different animal genera from the same location can have quite different tissue isotope compositions. However, for several reasons, Kohn’s model is limited in describing the variation within one species: (1) The model calculates the drinking water amount by subtracting the other input fluxes from total water demand, which accumulates all the errors from other input fluxes into that of drinking water. In addition, the amount of drinking water cannot directly reflect the influence of ambient conditions. (2) The model relates the total water demand only to the weight of animals and a genus-specific water economy index, which cannot reflect the seasonal change of total water demand. (3) The model does not consider lactation, which is a major output flux of O especially in dairy cows, and which requires an equivalent input flux. (4) Finally, δ^18^O of feed moisture is assumed to be equilibrated with δ^18^O of atmospheric humidity, while the δ^18^O in feed moisture in fact will change diurnally and thus grazing time and feeding strategy will also influence δ^18^O of ingested feed moisture.

For the reasons given above, we extended the Kohn model (we will refer to it as Munich Kohn model or MK model) as follows: (1) Drinking water intake was estimated directly based on water demand and water provision in the feed. The total water demand thus changed with temperature and relative humidity on a daily basis and the milk production of cows can be considered. (2) The body water turnover was considered by adding the O input fluxes to the body water from the previous day. (3) The different keeping conditions (housing versus pasturing) and feeding strategies were considered. For grazing animals this includes consideration of the convolution of both diurnal rhythms, that of grazing and that of δ^18^O in feed. In consequence, only the general principle of creating an animal’s mass balance for water and O were taken from the original Kohn model while the details had to be modified.

By knowing δ^18^O of body water from the MK model, the δ^18^O in hair was predicted and compared to the five-year variation of δ^18^O in hair of a domestic suckler cow subject to significantly different seasonal keeping strategies. The MK model will be useful to validate a reported origin and feeding strategy of domestic animals by measuring δ^18^O in hair.

## Materials and Methods

### Keeping and feeding strategy

The sampling was performed at Grünschwaige Experimental Station, Germany (48°23′N, 11°50′E), where a grazing experiment^24^ has existed between 1999 and 2012. The animals were kept on an organic farm approved by Naturland e.V., whose regulations also covered the standards of Canadian Council on Animal Care^25^. No other actions than necessary for animal husbandry were carried out. A Limousin suckler cow was selected from a herd of about 10 animals, which was in its second gestation and had a body weight of 637 kg at first hair sampling. The cow suckled a calf in the periods of 09 Dec 2000~22 Nov 2001, 11 Jun 2002~30 Mar 2003, 26 May 2003~14 Jan 2004 and 12 May 2004~07 Jan 2005. The herd was on paddock No. 8, 11, or 13; for paddock properties see Schnyder *et al.* ^24^; for an overview of temporal changes in keeping conditions, lactation periods and sampling events see Supplementary Fig. S1).

The animals remained entirely at pasture during grazing seasons and during this time they did not have access to housing and did not receive any supplements except for minerals. During winter, the herd was kept in an open-front free stall (length 55 m, height of the open front 3.75 m; 12 m depth of the stall including the feeding table at the open front) with additional eave and ridge ventilation. A mixture of silage and hay, which came from the same farm, was fed during the stall period. In contrast to fresh grass, silage water after extraction from the silo is close to drinking water because the mown grass equilibrates with the soil water during wilting (−9.1 to −12.9‰, Sun *et al.* ^17^). The silage and hay was provided in the morning and remained on the feeding table until the next morning when any remaining orts were removed. The silage was taken to the stall from an open-front, drive-in silo, in which the silage face was exposed to air for about one day before feeding.

The crude protein contents in the feed dry matter were 15.3 ± 1.4% (grass, n = 16) and 12.9 ± 0.45% (silage + hay, n = 4) on average for grazing seasons and stall seasons. In all seasons, the cow had free access to drinking water which was taken from local ground water.

### Hair and water sampling and isotope analysis

Sampling carried out during animal weighing on the farm comprised only tissue (hair) that was dead prior to sampling (approved by Technische Universität München). At the beginning and end of the grazing seasons of 2001–2004, hair was collected from the tail switch of the cow (for sampling dates see Supplementary Fig. S1). The hair was cleaned and cut into segments of 1 cm length (for details, see Auerswald *et al.* ^8^). These segments were packed in silver cups (4 to 6 mm) and analyzed by the pyrolysis method in a continuous flow system with an elemental analyzer (EURO EA 3028; Euro Vector, Milan, Italy) interfaced to an IsoPrime isotope ratio mass spectrometer (GV Instruments, Manchester, UK). Each sample was measured against a CO-reference gas calibrated against a secondary isotope standard (benzoic acid, IAEA-601). Stable isotope ratios (^18/16^O) are given in δ notation and expressed in per mil:

$${\rm{\delta }}(\permil )=({{\rm{R}}}_{{\rm{sample}}}/{{\rm{R}}}_{{\rm{standard}}}-1)\times 1000\permil ,$$

where R is the ratio of heavy to light isotopes. Standard is VSMOW (Vienna Standard Mean Ocean Water). For brevity, we only write δ with a subscript to indicate the substance under focus, thus δ_hair_ denotes O isotope composition in hair.

Air vapor (n = 86), precipitation (n = 90), groundwater (n = 87), stem water (n = 162) and soil water at 7 cm (n = 160) was sampled every one or two weeks at Grünschwaige Experimental Station from 2006 to 2012. Stems were not sampled on days when dew or rain was adhering to the grass. Furthermore, days with frozen or snow covered soil were excluded because animals were in the stalls under these conditions. The samples were then stored in a −18 °C freezer until water extraction by cryogenic vacuum distillation (2 h at 80 °C). δ^18^O in water samples was measured using Cavity Ring-Down Spectroscopy (CRDS, Picarro, USA). Each sample was measured repeatedly (more than four injections) and the values of the last two measurements were averaged (SD for repeated measurement was ±0.1‰). After every 20 to 25 samples, two laboratory water standards, derived from local deionized tap water by evaporation/condensation processes and covering the range of the isotope compositions of the samples, were measured for possible drift correction and normalizing results to the VSMOW scale. The laboratory standards were previously calibrated against V-SMOW, V-GISP and V-SLAP (from IAEA) using the same analytical procedure as used in sample analysis.

### Position-time assignment of δ_hair_ segments

To convert the position along the hair to a certain date, the hair growth rate needs to be known. Corresponding sections on hair shafts from subsequent samplings, which had grown at the same time, were localized by statistical isotopic pattern matching^26^. The hair growth rate was then given as length of the newly grown part of the younger hair per time interval between successive sampling dates. Hair growth rates were additionally validated by evaluating the ^13^C and ^15^N pattern of replicate hairs in the same way. Hair growth rate was 0.76 mm/d and varied little (slightly lower during stall periods and slightly decreasing with increasing age of the animal; for details, see Auerswald *et al.* ^8^). Hair growth rate was then used to interpolate between two subsequent sampling intervals and to assign a growth period to each 1 cm length segment of hair. On average, 1 cm of hair corresponded to a growth period of 13.2 d. Hairs usually comprised more than one year while the sampling interval was 0.5 yr in most cases (Supplementary Fig. S1). Thus, two to three hair segments from different hairs covered the same growth period.

### Modelling

#### General principle

The δ^18^O of body water **(**δ_bw_) results from the quantities (M in mole/d) and isotopic compositions **(**δ in ‰) of O input fluxes (air O uptake, air water vapor into the lungs, chemically bound O in feed, feed moisture and drinking water) and output fluxes (CO_2_ production, fecal water, milk water, orally and nasally exhaled respiratory water, O contributing to organic products, sweat water, transcutaneous water vapor, urea and urinary water), which must balance^2^:

1 $$\begin{array}{l}({{\rm{M}}}_{{\rm{air}}}\times {{\rm{d}}}_{{\rm{air}}}+{{\rm{M}}}_{{\rm{vapor}}}\times {{\rm{d}}}_{{\rm{vapor}}}+{{\rm{M}}}_{{\rm{bO}}}\times {{\rm{d}}}_{{\rm{bO}}}+{{\rm{M}}}_{{\rm{fw}}}\times {{\rm{d}}}_{{\rm{fw}}}+{{\rm{M}}}_{{\rm{dw}}}\times {{\rm{d}}}_{{\rm{dw}}})/{{\rm{M}}}_{{\rm{inputO}}}\\ \quad =\,({{\rm{M}}}_{{{\rm{CO}}}_{2}}\times {{\rm{d}}}_{{{\rm{CO}}}_{2}}+{{\rm{M}}}_{{\rm{fecal}}}\times {{\rm{d}}}_{{\rm{fecal}}}+{{\rm{M}}}_{{\rm{milk}}}\times {{\rm{d}}}_{{\rm{milk}}}+{{\rm{M}}}_{{\rm{oral}}}\times {{\rm{d}}}_{{\rm{oral}}}+{{\rm{M}}}_{{\rm{nasal}}}\times {{\rm{d}}}_{{\rm{nasal}}}\\ \quad \,\,\,+{{\rm{M}}}_{{\rm{P}}}\times {{\rm{d}}}_{{\rm{P}}}+{{\rm{M}}}_{{\rm{sweat}}}\times {{\rm{d}}}_{{\rm{sweat}}}+{{\rm{M}}}_{{\rm{cutan}}}\times {{\rm{d}}}_{{\rm{cutan}}}+{{\rm{M}}}_{{\rm{urea}}}\\ \quad \,\,\,\times {{\rm{d}}}_{{\rm{urea}}}+{{\rm{M}}}_{{\rm{urinary}}}\times {{\rm{d}}}_{{\rm{urinary}}})/{{\rm{M}}}_{{\rm{outputO}}}\end{array}$$

The amount of metabolic water results from the chemically bound H in digested feed^2^ minus the amount of H required for urea production; its O originates from the chemically bound O in feed and from air O. A description of all variables, their abbreviations and units is given in Supplementary Table S1.

In order to consider the turnover of body water in the MK model, the body water at day i was calculated by adding the O input fluxes to the body water of day i-1:

2 $$\begin{array}{l}({{\rm{M}}}_{{\rm{inputO}}}\times {{\rm{\delta }}}_{{\rm{inputO}}}+{{\rm{M}}}_{{\rm{bw}},{\rm{i}}-1}\times {{\rm{\delta }}}_{{\rm{bw}},{\rm{i}}-1})/({{\rm{M}}}_{{\rm{inputO}}}+{{\rm{M}}}_{{\rm{bw}},{\rm{i}}-1})\\ \quad =\,({{\rm{M}}}_{{\rm{outputO}}}\times {{\rm{\delta }}}_{{\rm{outputO}}}+{{\rm{M}}}_{{\rm{bw}},{\rm{i}}}\times {{\rm{\delta }}}_{{\rm{bw}},{\rm{i}}})/({{\rm{M}}}_{{\rm{outputO}}}+{{\rm{M}}}_{{\rm{bw}},{\rm{i}}})\end{array}$$

Fecal water, milk water, sweat water and urinary water are derived from body water without fractionation^2^. Thus, their isotopic composition was replaced by δ_bw,i_. The output fluxes subject to fractionation (CO_2_ production, O in organic products, urea, respiratory and transcutaneous water vapor) resulted from δ_bw,i_ + ε, where ε denotes the isotopic fractionation between an output flux and body water. Therefore δ_bw,i_ is solved:

3 $$\begin{array}{rcl}{{\rm{\delta }}}_{{\rm{bw}},{\rm{i}}} & = & ({{\rm{M}}}_{{\rm{inputO}}}\times {{\rm{\delta }}}_{{\rm{inputO}}}+{{\rm{M}}}_{{\rm{bw}},{\rm{i}}-1}\times {{\rm{\delta }}}_{{\rm{bw}},{\rm{i}}-1}-{{\rm{M}}}_{{\rm{oral}}}\times {{\rm{\varepsilon }}}_{{\rm{oral}}}-{{\rm{M}}}_{{\rm{nasal}}}\times {{\rm{\varepsilon }}}_{{\rm{nasal}}}\\  &  & -{{\rm{M}}}_{{\rm{cutan}}}\times {{\rm{\varepsilon }}}_{{\rm{cutan}}}-{{\rm{M}}}_{{{\rm{CO}}}_{2}}\times {{\rm{\varepsilon }}}_{{{\rm{CO}}}_{2}}-{{\rm{M}}}_{{\rm{p}}}\times {{\rm{\varepsilon }}}_{{\rm{p}}})/({{\rm{M}}}_{{\rm{inputO}}}+{{\rm{M}}}_{{\rm{bw}},{\rm{i}}})\end{array}$$

At the same time, the water mass balance of an animal was assumed to be zero,

4 $${{\rm{M}}}_{{\rm{input}}{{\rm{H}}}_{2}{\rm{O}}}-{{\rm{M}}}_{{\rm{output}}{{\rm{H}}}_{2}{\rm{O}}}=0,$$

As well as the oxygen mass balance:

5 $${{\rm{M}}}_{{\rm{input}}{\rm{O}}}-{{\rm{M}}}_{{\rm{output}}{\rm{O}}}=0,$$

The details of calculation of the MK model are given in Supplementary Tables S2 and S3 together with the sources from which the equations were taken. These equations were selected to cover a wide range of conditions. For example, the equation for respiration was derived for ambient temperatures between −12 °C to +40 °C^27^. In extreme cases not covered by these sources and especially in cases of other domestic ruminants like sheep or goat these equations should be replaced by more appropriate ones.

#### Input fluxes

Air O uptake and water vapor intake were calculated based on body weight, temperature and relative humidity according to Kohn^2^. The amount of chemically bound O from feed results from the chemical composition of the feed, digestibility and dry matter intake. The dry matter intake was calculated from metabolizable energy, which was determined by milk production, days in gravidity and body weight. The weight was measured at every occasion of movement from stall to pasture and back. Body weight was linearly interpolated between two measurements. In case of high yielding cows a considerable change in weight may occur especially during early stages of lactation due to the melt down of body reserves. The metabolism of body reserves was not considered because O released from the degradation of body lipids and proteins contributes very little to the total O intake. In case that 1 kg d^−1^ of body reserves would be metabolized (not including the export to milk, which is not relevant for the water balance of a cow) and this would contribute only 0.1% to the total O intake, which is irrelevant quantitatively but also isotopically.

Feed moisture was calculated as sum of internal water and adhering water from intercepted rain and soil water from dew rise. The internal water represented grass water in leaf and stem in grazing seasons and silage and hay water in stall seasons. Water contents of fresh grass were obtained from long-term measurements during the growing seasons at Grünschwaige Experimental Station. For the low canopy height of our pasture (compressed height of the sward was controlled to be 7 cm) we estimated that most of the intake comprised leaves (90%) and the remainder (10%) being (pseudo-) stems in grazing seasons. In the Kohn model, drinking water was calculated by subtracting the other input fluxes from total water demand, which did not reflect the seasonal variation of drinking water intake. We used the models described by Cardot *et al.* ^28^ for the stall period and Sun *et al.* ^15^ for the grazing period to estimate drinking water intake as a function of ambient conditions (relative humidity, daily average temperature and precipitation), milk production, and soil water storage (during grazing). The Sun model was modified to consider the influence of body weight (an increase of 1 kg body weight causes 0.1 kg increase in drinking water^14^). Sun’s model also yielded the amounts of intercepted rain water and soil water from dew rise, which adheres to the grazed grass.

#### Output fluxes

The estimation of CO_2_ production depended on the ingested feed, digestibility, an energy extraction factor, and the amount of O flowing into organic products (milk, growth). Kohn^2^ assumed the energy extraction factor to be 0.9 in herbivores, which is not applicable for cows because, in contrast to monogastric herbivores, ruminants lose a considerable amount of energy as methane. Metabolizable energy is thus only 82% of digestible energy^29^. From an isotope point of view, the distinction of fecal, urinary and sweat water appeared unnecessary, given that these body fluids are formed from body water without obvious fractionation^30–32^. Hence these excretions were considered together. Their amount was calculated as the difference between total water intake and all other water losses. The respiratory water was estimated from air flow through the lungs according to Stevens^27^ who considered air temperature. We assumed that two thirds of the respiratory water output was from oral expiration and one third was from nasal expiration. The equation by Stevens^27^ covers a range in temperature between −12 °C to +40 °C and thus also considers panting, but in our case panting did not occur because the temperature–humidity index was always lower than 78, which is considered the threshold above which a cow starts to pant^33^. Calves suck usually 5–10 kg/d from suckler cows^34^; following Häusler *et al.* ^35^ we used a linear decrease from birth (10 kg/d) to weaning (5 kg/d) at a rate of 0.02 kg/d^2^. The transcutaneous vapor was estimated from ambient conditions using the model built by Maia *et al.* ^36^. Further model components came from^37–40^.

#### δ^18^O of input and output fluxes

δ^18^O of air O utilized in the lungs was set to a typical value of 15.1‰, which is caused by the fractionation during O uptake by the lungs^2^. The δ^18^O of air vapor intake into the lungs was estimated from a long-term relation between average daily temperature and δ^18^O of vapor determined between 2006 to 2012 at the research site (in total 80 measurements; δ_vapor_ = 0.34 × T_av_ − 21.52; R^2^ = 0.49; *p* < 0.05).

In the Kohn model the feed moisture is considered to be equilibrated with air vapor. However, the δ^18^O of leaf water in grass changes seasonally and diurnally. To account for this change, MuSICA (Multi-layer Simulator of the Interactions between a vegetation Canopy and the Atmosphere) was parameterized for the research pasture and validated with six years of eddy covariance measurements and with the δ^18^O data for soil, stem and leaf water average^41^. MuSICA is a process-based, isotope-enabled model that simulates the exchanges of mass (water, CO_2_) and energy in the soil-vegetation-atmosphere continuum as well as the isotopic composition of ecosystem water pools. For details of the MuSICA parameters and validation see Ogée *et al.* ^42^. MuSICA can be run in 30-min time steps over multiple years or decades^42, 43^. The range of diurnal variation in 1-hr steps is given in Supplementary Fig. S2. We assumed there were four feeding peaks within a day (6:00, 11:00, 15:15 and 21:30)^44^ and the feed intake during these four periods to be equal to obtain the mean δ^18^O of the ingested leaf water for each individual day. In contrast to leaves, the water in stems is not enriched by transpiration. δ^18^O of stem water was set equal to the long-term (2006 to 2012) monthly isotope measured data of stem water at the research site. δ^18^O of precipitation was also used for that of adhering water. The δ^18^O of winter feed (silage and hay) water was calculated based on the model built by Sun *et al.* ^17^ and the assumption that the silage was exposed to air for 24 h. The seasonal change of feed bound δ^18^O was not measured. For simplicity it was assumed to be the same as in its main constituent cellulose. δ^18^O in cellulose during grazing seasons was estimated from δ^18^O of stem water and leaf water during the previous 30 d according to Cernusak *et al.* ^45^. In stall seasons it was assumed to be equal to the average growing-season cellulose O. The δ^18^O of drinking water was derived from the long-term measurement of ground water (88 measurements), which was almost constant.

The δ^18^O of organic products (milk constituents, fetal growth) was determined from the fractionation between body water and protein, which was assumed to be 15‰ according to results obtained for cows^46^ and woodrats^23^, ^46^. The δ^18^O of other output fluxes was determined from δ_bw_ and the specific fractionation values between output fluxes and body water for a herbivore following Kohn^2^.

#### Hair

δ_hair_ was predicted from δ^18^O of body water. O’Gady *et al.* ^47^ report a fractionation of 16.4‰ while Podlesak *et al.* ^23^ give a range between 13‰ and 17‰, which is similar to the range found between milk water and milk protein (14 to 16‰^46^). The reason for this wide range is unknown. Hence we used a mean value (15‰)^23^ in the first simulations. In a final simulation we treated the body water-keratin fractionation as a fitting parameter to obtain the best value under our conditions.

### Input data

The input data of the model contained parameters including weather data, δ^18^O of vapor, of precipitation, of stem water, of soil water, and of groundwater, MuSICA output, soil properties, feed properties, and animal properties. The weather data (average daily temperature, minimum temperature, relative humidity, precipitation, vapor pressure) were obtained from the Munich airport meteorological station (about 3 km from the grassland site) operated by the German Weather Service. From δ^18^O of water measured in Grünschwaige Experimental Station the long-term biweekly δ^18^O of precipitation and of stem water were determined and used as estimates of δ^18^O of intercepted water and of stem water; the long-term average δ^18^O of ground water was used as the δ^18^O of drinking water; long-term daily vapor data were used to evaluate the relationship between temperature and δ^18^O of vapor.

The MuSICA model delivered the δ^18^O of leaf water on an hourly basis. Soil parameters were taken from Schnyder *et al.* ^24^ and plant available water was modelled on a daily basis, following these authors. The water contents of silage and hay (n = 137) were recorded during the experiment. The digestibility of feed was determined according to the fecal nitrogen method, which proved to be the best method under the experimental conditions^48^. The crude protein needed for this calculation was obtained from the nitrogen content of the grass.

### Statistics

Simple linear regressions were used to analyze the relation between two parameters. Paired t-test was used to compare the difference between average δ_hair_modelled_ and δ_hair_measured_. The root mean squared error (RMSE) was used to quantify mean deviations between prediction and measurement. In order to investigate how much the variation of different O sources and ambient conditions affected the variation of δ_hair_, individual influxes or ambient conditions were set constant to their long-term mean. The change in variation compared to the full model reflected the influence of this parameter. This approach can only quantify the influence of the variation but it does not reflect how much an individual flux causes the body water to change. This was quantified by calculating isofluxes, which are given by the difference in δ^18^O between a flux and that of the body water multiplied by the daily flux rate. The average relative isoflux (C_average_j_) of flux j is thus given by:

6 $${{\rm{C}}}_{{\rm{average}}\_{\rm{j}}}={{\rm{M}}}_{{\rm{j}}}\times |{{\rm{\delta }}}_{{\rm{bw}}}-{{\rm{\delta }}}_{{\rm{i}}}|/{\rm{\Sigma }}({{\rm{M}}}_{{\rm{j}}}\times |{{\rm{\delta }}}_{{\rm{bw}}}-{{\rm{\delta }}}_{{\rm{j}}}|)\times 100$$

where M_j_ and δ_j_ are the O amount (mole) and δ^18^O of the flux j, respectively. δ_bw_ is the δ^18^O of body water at day i.

Significance, if not explicitly stated, always refers to *p* < 0.05. Data are presented as mean values ± standard deviation.

## Results

### Relations between δ_hair_measured_ and ambient moisture sources (vapor, precipitation, soil, plants)

**Figure 1:**
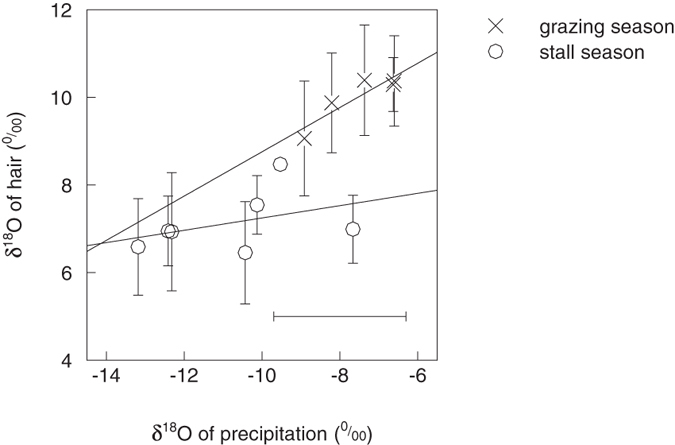
Relationships between monthly averages of measured δ_precip_ and δ_hair_measured_. Crosses (grazing season) and circles (stall season) represent the multi-year averages for each specific month when the cow was entirely either in stall or on pasture (90 and 97 hair data and 53 and 27 precipitation data in grazing season and stall season, respectively). The R^2^ of the linear regressions are 0.87 (*p* < 0.01) and 0.13 (*p* = 0.38), respectively in grazing and stall seasons, and 0.65 (*p* < 0.01) when taken together. Error bars denote standard deviations. Note that for simplification the horizontal error bar represents only the average of the monthly standard deviations.

δ_precip_ had significant seasonal variation, with monthly averages ranging from −13.2 to −6.6‰ (Fig. 1). It was higher in grazing seasons (−7.2 ± 2.2‰) than in stall seasons (−10.2 ± 2.0‰). δ_hair_measured_ (varying from 6.5 to 10.4‰) also was higher in grazing seasons (10.0 ± 1.1‰) than in stall seasons (7.1 ± 1.0‰) yielding about the same difference between seasons as precipitation. For the grazing seasons, averages of δ_hair_measured_ and δ_precip_ had a significant linear relationship on a monthly scale (*p* < 0.01, R^2^ = 0.873, N = 5) whereas the relationship was not significant for stall seasons (*p* = 0.38, R^2^ = 0.133, N = 7).

Measured leaf water was significantly enriched compared to precipitation, soil water and stem water because of plant transpiration (Supplementary Fig. S2). The δ^18^O of soil water, stem water, leaf water and precipitation were all positively related while the relation between leaf and precipitation was not significant (Supplementary Fig. S2). The R^2^ between stem water and soil water was highest (R^2^ = 0.77) and values were close to the 1:1 line.

### Dependence of δ_hair_measured_ on temperature, humidity and VPD

**Figure 2:**
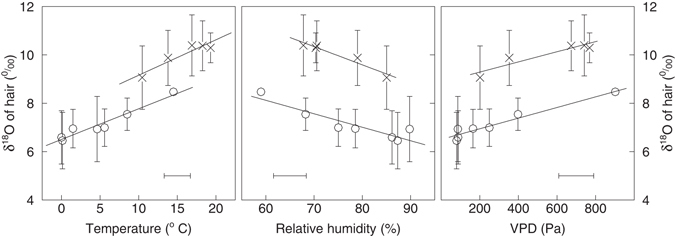
Relationships between monthly averages of δ_hair_measured_ and air temperature, relative humidity and vapor pressure deficit (VPD). Crosses (grazing seasons) and circles (stall seasons) represent the averages from 2000 to 2004 for each specific month where the cow was entirely either in stall or on pasture (90 and 97 hair data and 909 and 733 daily ambient conditions data in grazing season and stall season, respectively). The R^2^ of a linear regression between temperature (or relative humidity or VPD) and δ_hair_ in grazing and stall seasons was always significant (*p* < 0.01) (for temperature: R^2^ = 0.65 and R^2^ = 0.70, respectively; for relative humidity: R^2^ = 0.93 and R^2^ = 0.82, respectively; for VPD: R^2^ = 0.87 and R^2^ = 0.94, respectively). Error bars denote standard deviations. Note that for simplification the horizontal error bars represent only the average of the monthly standard deviations.

In grazing seasons the average monthly temperature (15.1 ± 4.6 °C) was significantly higher than that in stall seasons (3.3 ± 5.4 °C). However, there was no significant difference in monthly relative humidity between grazing seasons (73 ± 12%) and stall seasons (81 ± 13%). Linear relations between temperature (or relative humidity or vapor pressure deficit) and δ_hair_measured_ were all significant, but the relations differed between grazing and stall seasons (Fig. 2). During the grazing season the hair was more enriched than in the stall season, even when temperature, relative humidity or vapor pressure deficit were identical.

### Modelled O input and output fluxes through the cow’s body water

**Figure 3:**
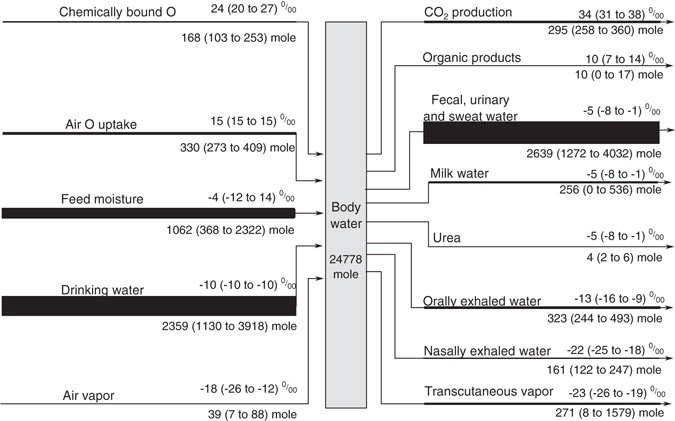
Modelled daily O input and output fluxes through the body water of a suckler cow. Values below and above the lines denote the mean and range (in parentheses) of the flux rates (mole d^−1^) and δ^18^O (‰) for the years 2000 to 2004, respectively. The fluxes are ordered according to δ^18^O. Line width is proportional to flux rate. The mean and range of δ^18^O in body water is identical to fecal, urinary and sweat water.

On average, the modelled drinking water (2359 mole d^−1^) and fecal, urinary and sweat water (together 2639 mole d^−1^) were the highest input and output fluxes, respectively, while the modelled air vapor intake into the lungs (39 mole d^−1^) and the urea excretion (4 mole d^−1^) were the lowest fluxes (Fig. 3). Of the input fluxes, the range of drinking water intake was highest, followed by feed moisture. Among the output fluxes, the amount of fecal, urinary and sweat water varied most, followed by transcutaneous vapor, milk water, orally exhaled water and nasally exhaled water, while all other output fluxes had a comparably narrow range.

**Figure 4:**
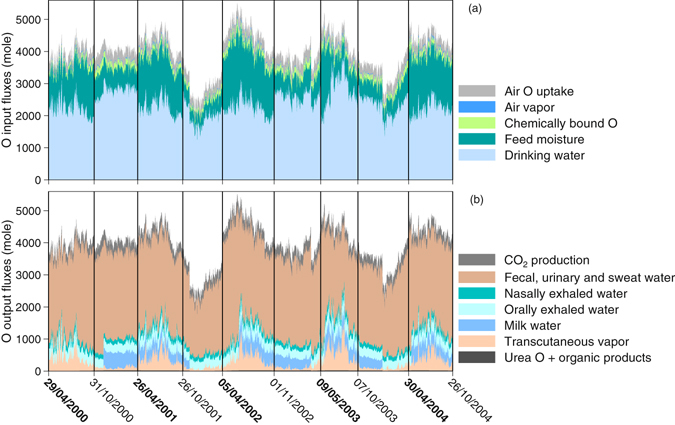
Modelled O amounts of input (**a**) and water output fluxes (**b**) during five years. Vertical lines and time labels show times of diet shift (bold labels indicate start of grazing; normal labels indicate start of stall seasons).

The main variations of total input and output fluxes were caused by the fluctuations of drinking water intake and fecal, urinary and sweat water (Fig. 4), which were driven by ambient conditions (mainly temperature but also precipitation and soil moisture content). The amount of feed moisture was lower in stall seasons than that in grazing seasons, which was compensated by higher consumption of drinking water in the stall (Fig. 4). There was an exception in the grazing season of 2003: the amount of feed moisture was low because it was an exceptionally dry summer with insufficient grass growth; it was necessary to supplement grazed grass with hay during this grazing season. The contribution of feed moisture to the total input flux was 33.3 ± 7.5% during grazing seasons and 16.8 ± 1.6% during stall seasons (Supplementary Table S4). The proportions contributed by drinking water were 54.0 ± 7.9% in the grazing seasons and 68.1 ± 2.6%, in the stall seasons. Both sources thus contributed the largest share of input fluxes in both seasons. In contrast, air O uptake, air vapor intake into lungs, and chemically bound O of feed contributed relatively little to the O input fluxes (grazing seasons: 7.7 ± 0.5%, 1.1 ± 0.2%, and 3.9 ± 0.5%, respectively; stall seasons: 9.5 ± 1.0%, 0.7 ± 0.2%, and 4.8 ± 0.5%, respectively).

Fecal, urinary and sweat water were the main O output fluxes in our model, contributing 64.3 ± 7.5% during grazing seasons and 70.6 ± 3.4% during stall seasons to the total output flux. CO_2_ production, milk water, orally exhaled water, transcutaneous vapor, nasally exhaled water, urea and organic products contributed relatively little to O output fluxes (grazing seasons: 7.1 ± 0.5%, 6.6 ± 4.6%, 8.3 ± 0.8%, 9.4 ± 4.9%, 4.1 ± 0.4%, 0.1 ± 0.0% and 0.2 ± 0.1%, respectively; stall seasons: 8.8 ± 1.0%, 5.6 ± 5.1%, 8.2 ± 1.3%, 2.6 ± 1.8%, 4.0 ± 0.6%, and 0.1 ± 0.0% and 0.3 ± 0.1%, respectively).

**Figure 5:**
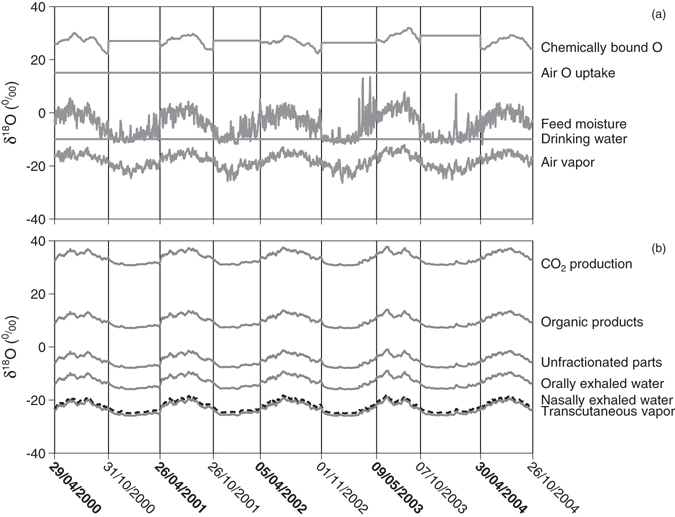
Modelled δ^18^O of input (**a**) and output fluxes (**b**) during five years. Vertical lines and time labels show times of diet shift (bold labels indicate start of grazing; normal labels indicate start of stall seasons).

Modelled δ^18^O of air O uptake was above ambient air O because of the fractionation during O uptake by the lungs. δ^18^O of air O uptake and of drinking water were constant (Fig. 5) while all other fluxes varied seasonally in δ^18^O. The range within other individual output fluxes was about 7‰ while the variation within other individual input fluxes was 7‰ or more. Feed moisture exhibited the largest variation (26‰) and, on average, it was more enriched during grazing seasons (−1.2 ± 2.8‰) than during the stall seasons (−8.0 ± 3.5‰). Modelled δ^18^O of chemically bound O was the most enriched input flux (grazing seasons: 23.8 ± 1.9‰; for the stall seasons the average over the previous growing season was assumed) (Fig. 5). In warmer months, like July and August, the values were relatively higher than in other months. Modelled δ^18^O of air vapor (grazing seasons: −16.2 ± 1.6‰; stall seasons: −20.5 ± 1.9‰) also had significant seasonal variation following temperature.

The isotope compositions of all output fluxes were determined by that of body water and constant fractionations. In consequence, the modelled δ^18^O of all output fluxes exhibited the same fluctuations over time even though they differed in absolute values due to different fractionations (Fig. 5). Higher values appeared during warm seasons than during cold seasons and also the fluctuation was larger during grazing seasons (SD: 1.4‰) than during stall seasons (SD: 0.7‰). In decreasing order the average values of δ^18^O for CO_2_ production, organic products, unfractionated fluxes, orally exhaled water, nasally exhaled water and transcutaneous vapor were 35.1‰, 11.5‰, −3.6‰, −11.6‰, −20.6‰ and −21.6‰ in grazing seasons and 31.4‰, 7.9‰, −7.2‰, −15.2‰, −24.3‰ and −25.2‰ in stall seasons.

Three parameters, namely drinking water, feed internal water and ambient conditions influencing the animal, were the main sources of variation in δ_hair_ (Supplementary Fig. S3), while the other parameters had little influence on the variation. The variation in drinking water intake dampened the fluctuation of hair in whole years (by 0.2‰) and grazing seasons (by 0.4‰). Feed internal water explained more than half of the variation of δ_hair_ within years (1.3‰) and within the grazing seasons (0.9‰); however, it explained only a small part (0.06‰) of the variation in stall seasons (Supplementary Fig. S3). Ambient conditions influencing the animal also caused about half of the variation of δ_hair_ within years (equal to 1.1‰), within grazing seasons (0.5‰) and within stall seasons (0.4‰). Hence modelling showed that almost half (46% within years, 36% within grazing seasons and 52‰ within stall seasons; Supplementary Fig. S3) of the seasonal variation in body water resulted from the animal itself and not from the feed. This may be easily overlooked due to the similarity in the seasonal variation of feed and body water, both of which are exposed to and transpire in the same environment.

A different picture was apparent from the calculation of isofluxes (Table 1). Expired CO_2_, drinking water, air O uptake and chemically bound O had the largest influence on body water because of their large isotopic contrast to body water (Fig. 3). Except for expired CO_2_ and chemically bound O, these fluxes did not vary in δ^18^O and thus dampen the isotopic variation of body water. As a result of this dampening effect, body water of the animal varied less than feed moisture (Fig. 5) although the combined and synchronous effects of feed moisture and ambient conditions influencing the animal would suggest that body water varies more than feed moisture. The resulting synchrony of feed moisture and body water then caused the isoflux of feed moisture to become small (Table 1) despite its pronounced seasonal variation in amount and δ^18^O.

**Table 1:** Average relative isoflux contribution (%) to the change of δ^18^O in body water by different fluxes.

Flux	Whole year	Grazing seasons	Stall seasons
CO_2_ production	27	24	32
Drinking water	22	26	16
Air O uptake	14	11	18
Chemically bound O	10	8	13
Transcutaneous vapor	9	14	4
Nasally exhaled water	7	5	7
Orally exhaled water	6	5	5
Feed moisture	5	5	3
Air vapor	1	1	1
Organic products	1	1	1
Unfractionated output fluxes	0	0	0

The relative isoflux contribution depends on the isotopic spacing between the flux and the body water and the amount of the flux.

### Relationship between ambient conditions (humidity and temperature) and modelled input and output fluxes of O

**Figure 6:**
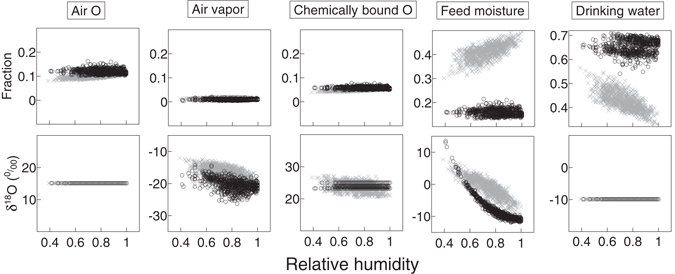
Relationships between relative humidity and modelled fractions of total O input fluxes (upper row) and modelled δ^18^O (lower row). Grey crosses and black circles represent grazing and stall seasons, respectively. Note that scaling of the y axes within a row is identical (0.4 for the upper row and 30‰ for the lower row) but the lower boundaries differ between panels.

The modelled input proportions of air O uptake, chemically bound O in feed, and feed moisture increased with increasing relative humidity in grazing seasons (Fig. 6, upper panels). This was compensated by a decreasing proportion of drinking water with increasing relative humidity. In the stall seasons, humidity had no influence on the amount of all input fluxes.

Modelled δ^18^O of air O uptake and drinking water was independent of relative humidity (Fig. 6, lower panels) while δ^18^O of air vapor and feed moisture decreased with relative humidity in both seasons. The δ^18^O of chemically bound O decreased only during the grazing seasons with increasing relative humidity, while there was no influence of actual humidity during stall seasons on bound O because it originated from the previous growing season.

**Figure 7:**
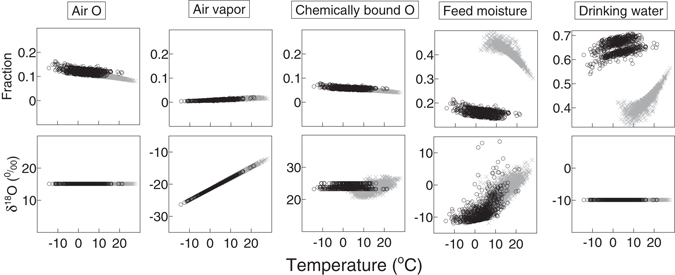
Relationships between temperature and the modelled fractions of total O input fluxes (upper row) and modelled δ^18^O (lower row). Grey crosses and black circles represent grazing and stall seasons, respectively. Note that scaling of the y axes within a row is identical (0.4 for the upper row and 30‰ for the lower row) but the lower boundaries differ between panels.

Modelled proportions of air O uptake, chemically bound O and feed moisture contributing to total water intake decreased in both seasons when the temperature increased (Fig. 7, upper panels), while there was an increasing trend for air vapor and drinking water with increasing temperature. The relations for air O uptake, vapor and chemically bound O almost overlapped in different seasons, but were pronouncedly separated for feed moisture and drinking water.

The δ^18^O of air O uptake and drinking water did not change with temperature (Fig. 7), while the modelled δ^18^O of air vapor was fully explained by temperature from which it was calculated. The modelled δ^18^O of chemically bound O in feed was not influenced by temperature in stall seasons but it increased with temperature in grazing seasons, although chemically bound O in feed was the result of growth during preceding days. This relation thus resulted from the higher probability of a warm day following warm days and a cold day following cold days. Modelled δ^18^O of feed moisture increased significantly more in stall seasons than in grazing seasons when temperature increased.

During stall seasons, there were two groups in the proportions of O input fluxes (especially pronounced for drinking water proportion in Figs 6 and 7). This separation was caused by the influence of weaning, which always happened during stall seasons (compare Supplementary Fig. S1). Drinking water demand then suddenly decreased because milk production terminated. During grazing seasons these two groups were not obvious because the cow suckled a calf most of the time (compare Supplementary Fig. S1) and the amount of milk gradually decreased with increasing age of the calf.

### Hair measurement and modelling

**Figure 8:**
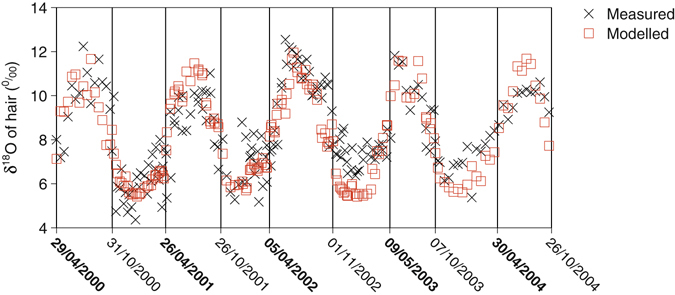
Measured and modelled δ^18^O of hair during five years. Black crosses and red circles represent measured and modelled values, respectively. Vertical lines and time labels show starting times of diet shift (bold labels indicate start of grazing; normal labels indicate start of stall seasons). Body water-keratin fractionation was obtained by fitting (14‰).

Modelled values were similar to measured values (Fig. 8). The RMSE between δ_hair_measured_ and δ_hair_modelled_ was 1.4‰ and δ_hair_measured_ was not significantly different from δ_hair_modelled_ (*p* > 0.05; paired t test). The model estimated the seasonal variation well: the δ_hair_measured_ and δ_hair_modelled_ almost simultaneously reached the minima in each stall season or the maxima in each grazing season and the modelled minima and maxima were close to the measured values.

## Discussion

A mechanistic model for predicting δ^18^O in body water turned out to be of high complexity despite the well documented, simple linear relationships between δ^18^O in body tissues and rain^9^. The mechanistic modelling required so many parameters that a practical use, e.g. for authenticity testing, is hardly conceivable. The value of such a model is threefold. It compiles our current understanding of the influences on body water δ^18^O. It allows understanding how the simple relation with δ^18^O in rain evolves (see discussion below). And finally, it allows deriving quantitative hypotheses (e.g. on the influences of body size or milk yield or soil) that then can be examined in controlled experiments for identification of gaps in our process understanding. The influences of such boundary conditions are so manifold and interacting that a sound hypothesis can hardly be created without such a model. The model further allows judging, which parameters must be measured or controlled in such an experiment for obtaining reliable results and for describing their range of validity. For instance: one of the most important single number in our modelling turned out to be the plant available water capacity of the soil, a parameter which is hardly ever measured or reported in animal studies. It entered our calculations in several places: (i) it influences water stress of the plant and thus δ^18^O in leaf water and chemically bound δ^18^O. (ii) It influences the mixing of rain water and thus stem water. (iii) It influences the amount of water adhering to the leaves from dewrise. (iv) And thus, it also influences the drinking water demand of the animals^15^.

A major disadvantage of such a complex model requiring many parameters is that it provides ample opportunity for parameter adjustment to improve the fit between prediction and measurement. We took great care not to adjust parameters but to use them as published or measured. E.g., we use the plant available water capacity as published (Schnyder *et al.* ^24^) without optimizing it within its range of uncertainty. Only the body water-keratin shift was optimized in a final step (see discussion below).

A second major disadvantage of such a complex model is that every parameter unavoidably carries some error; these are then combined in the model and interact. For instance, we used rainfall data from a station of the German Weather Service in 3 km distance. It is well known that rainfall can vary by a factor of two within a distance of only 1 km^49, 50^ although long-term rainfall should be identical within this distance. Our model offers the advantage of sensitivity testing to find out, which accuracies are needed for the individual parameters.

The measured δ_hair_ correlated with δ_precip_ during the grazing season, which seems to be in line with the finding of Ehleringer *et al.* ^9^ that δ_hair_ in humans correlates with δ^18^O in tap water on a regional scale, where tap water again reflects the regional variation in δ_precip_. Such a direct link, however, is true only to a small degree for cows, because δ_leaf_, which contributed most of the feed moisture during the grazing season, did not correlate with δ_precip_ (Supplementary Fig. S2). Only stem water and precipitation intercepted by the grass carry the isotopic information of precipitation but both contribute little to total water intake. Hence, the close correlation between δ_precip_ and δ_hair_ must originate from indirect relations. The δ_precip_ is closely linked with atmospheric humidity and temperature^51^. Simultaneously, about half of the seasonal variation of δ_hair_ was generated by the animal itself, which in turn was influenced by its ambient conditions (including temperature, air humidity); this created the apparently close relation between δ_hair_ and δ_precip_. Similarly, the seasonal variation found previously for hair, milk and tooth^2, 7, 52, 53^, and which apparently reflected the seasonal variation in feed moisture, resulted only partly from this variation, while the other part was caused by the animal. The detailed and mechanistic modelling of body water and δ_hair_ thus provided insights that easily could be overlooked in regression analyses due to the close correlations with some environmental water sources. An additional example of the seasonal variation caused by the animal itself under constant feeding conditions (total mixed ration and tap water) is given for milk data in Fig. S4 (right panel) in the Appendix.

The large contribution of the animal’s ambient conditions to the variation of δ^18^O in body tissues is advantageous when δ^18^O is used as an indicator of geographic origin because δ^18^O in body tissues is then less adulterated by the type of moisture uptake (e.g., the time of grazing or the moisture content of the feed). For the same reason, it is disadvantageous when δ^18^O in animal products is used to serve as an indicator of the production system (e.g. to distinguish between milk produced from fresh grass or silage) unless there is additional information available for the ambient conditions.

Lactation has a pronounced influence on drinking water uptake and this has been demonstrated in many previous studies^11, 12, 14, 28^. This influence became especially visible during the stall periods. Drinking water had the lowest δ^18^O among all water sources. Nevertheless, this pronounced variation in drinking water uptake caused by lactation was not evident in measured or modelled δ_hair_. The reason is that lactation also increases feed intake and thus the intake of enriched water during grazing seasons. Both of these lactation-induced changes compensate each other and thereby remove the influence of lactation. Similarly, during stall seasons, no net influence of lactation can result from an increased intake of drinking water because silage water and drinking water are similar in δ^18^O. The range of milk production of our suckler cow was narrow (10 kg d^−1^) while a much larger range can be found in dairy cows. Also for this much wider range the influence of lactation on δ^18^O in body water is negligible (for an example see Fig. S4 in the Appendix).

Ehleringer *et al.* ^9^ found that human hair O was enriched by about 22.7‰ compared to O in tap water, while in the case of our study the enrichment was about 3‰ less, although cows incorporate high proportions of highly enriched leaf water with their diet, in contrast to humans. Although Ehleringer *et al.* ^9^ did not report the average δ^18^O in the diet water; it is highly likely that the human diet, prepared mainly using tap water, is less enriched than the water of a cow diet, which consists mainly of fresh leaves. This discrepancy (higher values in humans despite lower values in diet) corroborates a major finding of our study, namely that a large part of the variation in body water results from the animal itself (e.g. transpiration, respiration and water demand). Water losses that cause enrichment of the body water average 23% of the water intake by humans^31^, while these losses comprised only 18% of the water intake of our cow. Thus, enrichment by respiration and transpiration can exert a larger influence in humans than in our cow.

The modelled δ^18^O of body water varied from −8 to −1‰, which was very close to the values found by Boner and Förstel^4^ in Germany (−7 to −1‰), and also the similarity of measured and modelled δ^18^O in hair suggests that the mechanistic MK model captures the most important influences. Major uncertainties are likely to be associated with (1) the estimation of δ^18^O in feed moisture and in (2) the estimation of δ_hair_ from the δ^18^O of body water:

(1) Uncertainty from estimation of δ^18^O in feed moisture: During summer there is a major uncertainty in δ^18^O of feed because of the unknown diurnal proportions of feed intake, likely to be affected by environmental variables (day length, temperature, rainfall). Based on MuSICA modelling, the diurnal range of δ^18^O of leaf water was about 7‰ on average (Supplementary Fig. S5), which agrees with other findings^54, 55^. Night-dominated grazing during a hot period may thus lead to a lower than average δ^18^O of ingested leaf water, while daylight grazing on a cool day may provide feed water above average δ^18^O. Secondly, we assumed a leaf to shoot ratio of feed of 0.9 according to our visual observations, whereas Kohn^2^ recommended a ratio of 0.5 for herbivores, and Durham^56^ reported a range from 0.56 to 0.84 for the Texas Coastal Prairie. Changing the leaf to shoot ratio in a sensitivity test showed that the mean offset between measurement and prediction would disappear for a leaf to shoot ratio of 0.3. Such low ratios are very unlikely in a canopy of 7 cm compressed sward height and do not comply with our visual observations; however, we are not aware of estimates that would describe the variation under a wide range of conditions.

During winter time, our knowledge and prediction abilities of feed moisture and chemically bound O are even more limited. We had assumed constant values for chemically bound O, while variations are highly likely because silage originates from different fields, from different days and from different harvesting conditions (e.g. time during day). However, it is not possible to predict which portions of silage from a silo are fed on a specific day or the properties these particular portions. Thus, during the stall period the MK model mainly reflected the variation created by the animal but not the variation originating from feed.

(2) Uncertainties of δ_hair_ estimation from the δ^18^O of body water: A mechanistic model for δ_hair_ was developed by Ehleringer *et al.*, Bowen *et al.* and O’Grady *et al.* ^9, 47, 57^ that shares the general principle of calculating body water from influxes and outfluxes, but it uses constants to describe these fluxes. The model assumes that δ^18^O in hair is derived from isotopic exchange with gut water during hydrolysis of dietary protein; gut water, in turn, results from the mixture of food water, drinking water and body water. This model was applied in humans, nonhuman primates, and woodrats. Cows are different to these species in having a four-compartment stomach involved in water absorption and remixing. Absorption and remixing make the prediction of gut water more complicated than in monogastric animals. For example, part of the drinking water may directly reach the omasum by bypass flow via the esophageal groove without mixing with the ruminal water, and some of the saliva can already be absorbed by the rumen^58^. The fraction of bypass flow and its drivers are unknown. For simplicity, a fractionation between body water and keratin derived from the average value in rodents (15‰)^23^ was used in our model. The best-fitted value was 14‰, which was close to the aforementioned estimate; however, the mechanisms underlying the fractionation between body water and hair should be investigated further for a range of species.

Recently, identification of food authenticity and geographical origin has become a crucial issue requested both by consumers and authorities because of the frequent global exchange of food. The European Union’s general food law (Regulation EC No. 178/2002) has made traceability compulsory for all food and feed businesses since 2005^59^. Multi-element stable-isotope ratio (SIR) analysis has been proved to be practical for this purpose^60^. However, the mechanisms of isotope flow in animals are still not fully understood, especially for δ^18^O. The application of δ^18^O is mainly based on the fundamental fact that δ^18^O in wild animal tissue is usually linearly related to δ^18^O in annual precipitation, which can be used to detect the geographical origin of animals along precipitation gradients^61^. Application of the MK model showed that this simple relation is the result of the interaction of many processes, and most of these can be manipulated in domestic animals. The δ^18^O in domestic animal tissues thus carries the convoluted information of geographic origin and animal husbandry and the MK model may be used for disentangling these influences (for an example of the application and validation of the MK model for milk see Fig. S4 in the Appendix).

There are two parts in the MK model: the estimation of δ^18^O of body water and the subsequent estimation of δ_hair_. The estimation of body water may be useful to identify if there is any fraud in the claimed origin of milk or meat by comparing the measured and modelled water O in them. However, it is indispensable to account for the seasonal variation. The seasonal δ^18^O variation of body water was 8‰ in our case. Chesson^62^ reported a regression between δ^18^O in milk and rain (δ_milk_ = 0.86 × δ_precip_ + 1.1), which implies that the regional difference in precipitation must be larger than 9‰ to override the seasonal variation when the time of production is not known.

The second part of the MK model, the estimation δ_hair_, also has some potential applications. Since the beginning of the Neolithic age about 10,000 years ago, humans have tried to influence the life cycle of domestic animals^7^. The isotopic study of the animal remains (such as hair) may shed some light on animal husbandry. The large number of variables influencing body water and hair, however, calls for a cautious interpretation, especially when ambient conditions are not known in detail.

In our case the MK model was applied in a temperate region where panting did not occur. Panting in heat stress conditions increases the orally exhaled water and thus causes an additional enrichment of the body water but other changes will happen simultaneously (increased transcutaneous vapor, increased drinking water uptake). The MK model offers the advantage to consider all changes in animal physiology simultaneously that are induced with increasing temperature (for an example see Fig. S6 in the Appendix, which shows that increasing transpiration, sweating and panting increases drinking water uptake and thus decreases the contribution of metabolic water to total water intake; the model results fit well to the data by Khelil-Arfa *et al.* ^63^, who quantified metabolic water to contribute about 5% under thermoneutral conditions (15 °C) and 4% under high-temperature (28 °C) conditions). A much larger effect of high temperatures as found in sub-tropical and tropical latitudes can be expected, however, from the differences in meteoric water, the difference in the diurnal adaptation of feeding and the differences in plant species composition. Under high temperature conditions animals will preferably graze at night^64^ when leaf water enrichment in minimal. Plant species composition changes from species with C_3_ photosynthesis to species with C_4_ photosynthesis, which have a considerably higher enrichment of δ^18^O in leaf water^65^ due to differences in water use efficiency and which may even exploit different sources of water^66^.

## Conclusions

The variation of δ_hair_ of a domestic cow results from the interplay of environment, animal physiology and feeding strategy. Temperature and relative humidity were significantly related to measured and modelled δ_hair_ in summer and winter seasons. Temperature and relative humidity not only influenced the feed (e.g. feed internal water composition) but also the animal itself, (e.g. drinking water intake). Modelling showed that almost half of the seasonal variation in body water resulted from the animal itself. This may be easily overlooked due to the similarity in the seasonal variation of feed and body water, both of which are exposed to and transpire in the same environment.

The mechanistic MK model explained well the variation between seasons and within seasons, although strong indications existed that the influences of animal behavior and animal physiology are still insufficiently understood for predicting δ^18^O in animal tissues. Nevertheless, the MK model allows accounting for animal husbandry and feeding strategy in domestic animals. This will foster our understanding of δ^18^O in animal products; it will allow identifying those management strategies for which δ^18^O in animal products can serve as a reliable proxy. Further tests of this model under different climatic and husbandry conditions in different regions are necessary for a wider application.

## Electronic supplementary material

Supplementary Tables and Figures
