# Supplementary material for: Model explanation of the seasonal variation of δ18O in cow (Bos taurus) hair under temperate conditions
Source: Sci Rep. 2017 Mar 23;7:320. doi: 10.1038/s41598-017-00361-y (PMC5428254; doi:10.1038/s41598-017-00361-y)
Supplement: Supplementary file 1 — Supplementary Tables and Figures [file 41598_2017_361_MOESM1_ESM.pdf]

Supplementary Information

Model explanation of the seasonal variation of δ18O in cow (Bos taurus) hair under temperate conditions

Guo Chen, Hans Schnyder, Karl Auerswald*

Table S1: Parameters, abbreviations and units used in this paper; part 1: fluxes.

Flux	abbreviation	unit
Intake of water adhering to feed	M_{adhere}	mole/d
O intake from air	M_{air}	mole/d
Intake of bound H in digested feed	M_{bH}	mole/d
Intake of bound O in digested feed	M_{bO}	mole/d
O in carbon dioxide flux	M_{CO2}	mole/d
Transcutaneous vapor flux	M_{cutan}	mole/d
Drinking water intake on dry day (when $(P_{i-1} + P_i)<0.02$ mm)	M_{dry}	mole/d
Drinking water intake	M_{dw}	mole/d
Fecal water loss	M_{fecal}	mole/d
Nasally exhaled water loss	M_{nasal}	mole/d
Orally exhaled water loss	M_{oral}	mole/d
O in organic products	M_p	mole/d
Exhaled water by respiration	M_{resp}	mole/d
Intake of feed internal water	M_{inner}	mole/d
Total O input flux	M_{inputO}	mole/d
Total water input flux	M_{input_H2O}	mole/d
Total O output flux	$M_{outputO}$	mole/d
Total water output flux	M_{output_H2O}	mole/d
Milk water	M_{milk}	mole/d
Sweat water loss	M_{sweat}	mole/d
Feed moisture uptake	M_{fw}	mole/d
Urinary water loss	$M_{urinary}$	mole/d
O flux with urea	M_{urea}	mole/d
Vapor O uptake by breathing	M_{vapor}	mole/d
Drinking water intake on wet day (when $(P_{i-1}+P_i)>2$mm)	M_{wet}	mole/d

Table S1, continued: Parameters, abbreviations and units used in this paper; part 2: Isotope compositions.

Water or oxygen source	abbreviation	unit
Winter feed before exposure to air	δ_0	‰
Water adhering to feed	δ_{adhere}	‰
O utilized in lungs	δ_{air}	‰
Body water	δ_{bw}	‰
Cellulose	δ_c	‰
Transcutaneous vapor	δ_{cutan}	‰
Bound O in feed	δ_{bO}	‰
Drinking water	δ_{dw}	‰
Feed moisture	δ_{fw}	‰
Hair	δ_{hair}	‰
Measured hair	$\delta_{hair\_measured}$	‰
Modelled hair	$\delta_{hair\_modelled}$	‰
Total O input flux	δ_{inputO}	‰
Leaf water	δ_{leaf}	‰
Nasally exhaled water	δ_{nasal}	‰
Orally exhaled water	δ_{oral}	‰
Total O output flux	$\delta_{outputO}$	‰
O in organic products	δ_p	‰
Precipitation	δ_{precip}	‰
Winter feed moisture in equilibrium with air	δ_{ss}	‰
Stem water	δ_{stem}	‰
O flux with urea	δ_{urea}	‰
Vapor in free air	δ_{vapor}	‰

Table S1, continued: Parameters, abbreviations and units used in this paper; part 3: Oxygen fractionations.

Fractionation	abbreviation	unit
between CO_2 and body water	ε_{CO2}	‰
between transcutaneous vapor and body water	ε_{cutan}	‰
between vapor and water in equilibrium	ε_{eq}	‰
Kinetic fractionation	ε_k	‰
between nasally exhaled water and body water	ε_{nasal}	‰
between oral exhaled water and body water	ε_{oral}	‰
between organic products and body water (15 ‰)	ε_p	‰
between carbonyl oxygen and water	ε_{wc}	‰

Table S1, continued: Parameters, abbreviations and units used in this paper; part 4: Other parameters.

Parameter	abbreviation	unit
Air flow through the lungs	A	L/d
Oxygen content in air (21 %)	C_{air}	%
Average relative contribution of flux j to the change of $\delta^{18}O$ in body water	$C_{average\_j}$	%
Carbohydrate content	C_c	%
Fat content	C_f	%
O conversion factor (0.00216 mole/KJ)	C_o	mole/KJ
Crude protein content	C_p	%
Digestibility	D	
Days in gravidity	d_g	d
Days in milk	d_m	d
Energy extraction efficiency	E_{ex}	
Energy used for heat production	E_H	KJ/d
Metabolizable energy	E_{met}	KJ/d
Energy used for mass production	E_P	KJ/d
Unit conversion factor L → mole (55.56 mole/L)	f_{mL}	mole/L
Unit conversion factor m^3 →mole (3 mole/m^3 vapor at body temperature)	f_{mcm}	mole/m^3
Unit conversion factor d →min (1440 min/d)	f_{mind}	min/d
Unit conversion factor d →s (86400 s/d)	f_{sd}	s/d
Unit conversion factor kJ →Ws (1000 Ws/kJ)	f_{WskJ}	Ws/kJ
Relative humidity	H	
Body weight	m_{animal}	kg
Dry mass intake of feed	m_{dry}	kg/d
Milk production	m_{milk}	kg/d
Oxygen extraction from air (0.2)	O_{ex}	
Proportion of oxygen atoms exchanging with medium water during cellulose synthesis	P_{ex}	
Precipitation at day i	P_i	mm/d
Relative plant available water	P_{rel}	mm/mm
Proportion of unenriched (source) water in tissue where cellulose synthesis is occurring	P_x	
Leaf to shoot ratio of feed	R	kg/kg
Animal surface area	S	m^2
Half-life of silage water	$t_{0.5}$	h
Exposure time of winter feed	$t_{exposed}$	h
Average daily temperature	T_{av}	°C
Minimum daily temperature	T_{min}	°C
Molar gas volume (25.5 L/mole at 38 °C)	V_m	L/mole
Water content of feed fresh matter	W_C	g/g

Table S2: Calculation methods for parameters of input fluxes. The equations were adjusted to common units and simplified where possible compared to original equations of the reported sources.

Parameter	Function	Source
A	$E_H \times C_o \times V_m / O_{ex} / C_{air} \times 100 =$ $E_H \times C_o \times 607$	Kohn[2]
E_H	$(5.6 \times m_{animal}^{0.75} + 1.6 \times 10^{-5} \times d_g^3 + 22 \times m_{milk}) \times f_{sd} / f_{WskJ}$ $= 484 \times m_{animal}^{0.75} + 1.4 \times 10^{-3} \times d_g^3 + 1900 \times m_{milk}$	DIN[63]
E_P	$2 \times (1.4 \times 10^{-3} \times d_g^3 + 1900 \times m_{milk})$	DIN[63]
E_{met}	$E_H + E_P$	Robbins[29]
m_{dry}	$E_{met} / (170 \times C_c + 400 \times C_f + 200 \times C_p) / D / E_{ex}$	Robbins[29]
M_{air}	$2 \times C_o \times E_H$	Kohn[2]
M_{bH}	$2 \times D \times E_{ex} \times m_{dry} \times (0.31 \times C_c + 0.6 \times C_f + 0.11 \times C_P)$	Kohn[2]
M_{bO}	$2 \times D \times E_{ex} \times m_{dry} \times (0.15 \times C_c + 0.02 \times C_f + 0.03 \times C_P)$	Kohn[2]
M_{fw}	$m_{dry} \times f_{mL} \times W_C / (1 - W_C) + M_{adhere}$	This study
M_{dw}	Grazing: (1) If $(P_{i-1} + P_i)<0.02$: $M_{dry} = (0.0011 \times T_{av}^3 + 8.8 + (-0.22 \times H + 13.3 - 0.0011 \times T_{av}^3) \times (P_{rel})^4 + (m_{animal} - 411) \times 0.1) \times f_{mL}$ $=0.061 \times T_{av}^3 - 1794.6 + (-12.22 \times H + 738.9 - 0.061 \times T_{av}^3) \times (P_{rel})^4 + m_{animal} \times 5.6$ (2) If $(P_{i-1} + P_i)>2$: $M_{wet} = (0.0013 \times T_{av}^3 + 4.4 + (-0.22 \times H + 17.7 - 0.0013 \times T_{av}^3) \times (P_{rel})^4 + (m_{animal} - 411) \times 0.1) \times f_{mL}$ $=0.072 \times T_{av}^3 - 2039.1 + (-12.22 \times H + 983.4 - 0.072 \times T_{av}^3) \times (P_{rel})^4 + m_{animal} \times 5.6$ (3) If $0.02<(P_{i-1} + P_i)<2$: $M_{dw} = (M_{wet} + M_{dry}) / 2$ Stall: $(1.53 \times m_{dry} + 1.33 \times m_{milk} + 89\times(1 - W_C) + 0.57 \times T_{min} - 0.3 \times P_i - 25.65) \times f_{mL}$ $= 85 \times m_{dry} + 74 \times m_{milk} - 4953 \times W_C + 32 \times T_{min} - 17 \times P_i + 3525$	Cardot et al.[28]; Sun et al.[15]
M_{input_H2O}	$M_{dw} + M_{fw} + M_{vapor} + M_{bH} / 2 - 2 \times M_{urea}$	This study
M_{inputO}	$M_{dw} + M_{fw} + M_{vapor} + M_{bO} + M_{air}$	Kohn[2]
M_{vapor}	$10^{(0.686+0.027Tav)} \times H \times A / 760 / V_m$	Kohn[2]
δ_{vapor}	$0.34 \times T_{av} - 21.52$	This study
δ_{leaf}	MuSiCA modelling	Ogee et al.[38]
δ_{ss}	$(\delta_{vapor} + \varepsilon_{eq} / H + (1 - H) / H \times \varepsilon_k) / (1 + \varepsilon_k / 1000 - 1 / H \times (\varepsilon_k + \varepsilon_{eq}) / 1000)$	Wen[64] Helliker et al.[65]
δ_{fw}	Grazing: $((\delta_{leaf} \times R + \delta_{stem} \times (1- R)) \times M_{inner} + \delta_{adhere} \times M_{adhere}) / M_{fw}$ Stall: $(\exp(-\ln(2)t_{exposed} / t_{0.5}))(\delta_0 - \delta_{ss}) + \delta_{ss}$	This study Sun et al.[17]
δ_c	$(\delta_{leaf} - \delta_{stem})(1 - P_x \times P_{ex}) + \delta_{stem} + \varepsilon_{wc}$ $= 0.58\ \delta_{leaf} + 0.42\ \delta_{stem} + 27$	Cernusak et al.[41]
δ_{inputO}	$(M_{air} \times \delta_{air} + M_{bO} \times \delta_c + (M_{inner} + M_{adhere}) \times \delta_{fw} + M_{dw} \times \delta_{dw} + M_{vapor} \times \delta_{vapor})/M_{inputO}$	This study

Table S3: Calculation methods for parameters of output fluxes. The equations were adjusted to common units and simplified where possible compared to original equations of the reported sources.

Parameter	Function	Source
M_{CO2}	$M_{outputO} – M_{output\_H2O} – M_{urea} – M_p$	This study
M_{cutan}	$85.18 \times e^{(Tav-24.92)/7.96} \times f_{sd} / (2500.7879 – 2.3737 \times T_{av}) \times S /18$<br>$= 408864 \times e^{(Tav-24.92)/7.96} / (2500.7879 – 2.3737 \times T_{av}) \times S$	Maia et al.[36]
$M_{fecal}+M_{urinary}+M_{sweat}$	$M_{output\_H2O} – M_{milk} – M_{cutan} – M_{oral} – M_{nasal}$	This study
M_{milk}	$(10 – 0.02 \times d_m) \times f_{mL}$<br>$=555.6 –1.1 \times d_m$	This study
M_p	$1.8 \times m_{milk} + 5.2 \times 10^{-7} \times d_g^3$	This study
M_{resp}	$f_{mcm} \times f_{mind} \times 0.0189 \times \exp(0.537 \times(2.966 + 0.00069 \times T_{av}^2 + 0.0218 \times T_{av}))$<br><br>$= 81.65 \times \exp(0.537 \times(2.966 + 0.00069 \times T_{av}^2 + 0.0218 \times T_{av}))$	Stevens[27]
M_{oral}	$2/3 \times M_{resp}$	Kohn[2]
M_{nasal}	$1/3 \times M_{resp}$	Kohn[2]
M_{urea}	$m_{dry} \times D \times E_{ex} \times C_p \times 0.06$	Kohn[2]
S	$0.09 \times m_{animal}^{0.67}$	McGovern et al.[66]

Table S4: Modelled contributions of each flux (%) to total amount of O input or output flux for the whole year, the grazing seasons and the stall seasons. The values are presented as mean ± SD.

	Contribution (%)	Whole year	Grazing seasons	Stall seasons
Input fluxes	Drinking water	60.3 ± 9.3	54.0 ± 7.9	68.1 ± 2.6
	Feed moisture	26.0 ± 10.0	33.3 ± 7.5	16.8 ± 1.6
	Air O uptake	8.5 ± 1.2	7.7 ± 0.5	9.5 ±1.0
	Chemically bound O	4.3 ± 0.7	3.9 ± 0.5	4.8 ± 0.5
	Air vapor	1.0 ± 0.3	1.1 ± 0.2	0.7 ± 0.2
Output fluxes	Fecal, urinary and sweat water	67.1 ± 6.8	64.3 ± 7.5	70.6 ± 3.4
	Orally exhaled water	8.2 ± 1.0	8.3 ± 0.8	8.2 ± 1.3
	CO_2 production	7.9 ± 1.1	7.1 ± 0.5	8.8 ± 1.0
	Transcutaneous vapor	6.4 ± 5.2	9.4 ± 4.9	2.6 ± 1.8
	Milk water	6.2 ± 4.9	6.6 ± 4.6	5.6 ± 5.1
	Nasally exhaled water	4.1± 0.5	4.1± 0.4	4.0 ± 0.6
	Organic products	0.3 ± 0.1	0.2 ±0.1	0.3 ± 0.1
	Urea	0.1 ± 0.0	0.1 ± 0.0	0.1± 0.0

Table S5: Overview of O input fluxes.

Input	Mean amount (mole)	Mean $\delta^{18}O$ (‰)	Range of $\delta^{18}O$ (‰)	Source of $\delta^{18}O$
Drinking water	2359	-10	-9.5 to -10.5	Measured
Leaf water intake	1015	0	-11 to 9	Measured and MuSICA modeled[37]
Silage water intake	594	-8	-12 to 14	Sun et al.[17]
Air O uptake	330	15.1	15.1 to 15.1	Kohn[2]
Chemically bound O	168	24	20 to 27	MuSICA modeled[37]
Stem water intake	113	-9	-13 to -7	Measured
Air vapor	39	-18	-26 to -12	Measured
Water adhering to leafs	32	-15	-15 to -2	From measured precipitation

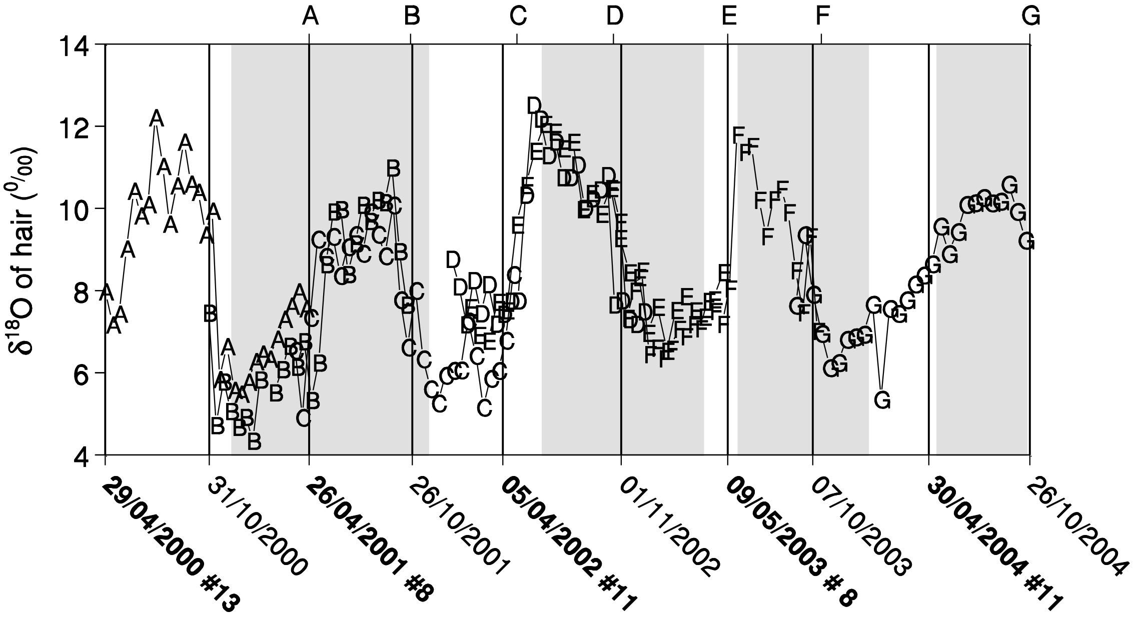

Fig. S1: The $\delta^{18}O$ of tail switch hairs and keeping conditions during five years. The upper x axis labels indicate the time of sampling tail hairs and the respective letters are used as markers for the $\delta^{18}O$ data of every 1-cm piece of hair from the root of the hair at the sampling date to the hair tip as detailed in Materials and Methods, section ‘position-time assignment of hair segment data’. Vertical lines and lower x axis labels show times of grazing/keeping shifts (bold labels followed by the paddock number indicate start of grazing; normal labels indicate start of stall seasons). Grey shaded areas denote periods when the cow fed a calf.

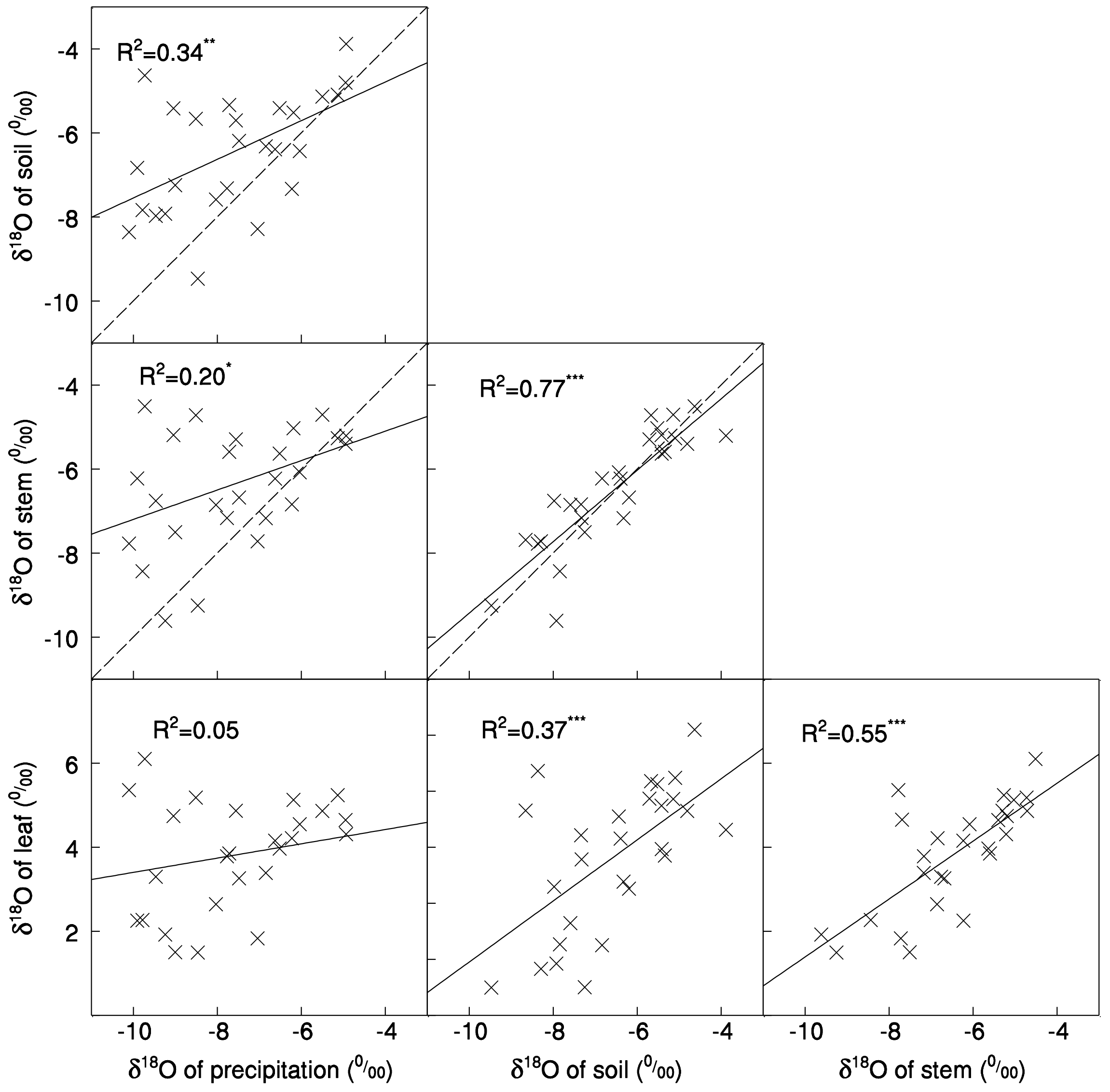

Fig. S2: Relation among average monthly $\delta^{18}O$ of soil water, stem water, leaf water and precipitation of measurements at midday from 2006 to 2012 (N=27 for each item). Solid and dashed lines represent linear regressions and 1:1 lines, respectively. * $p<0.05$, ** $p<0.01$, and *** $p<0.001$.

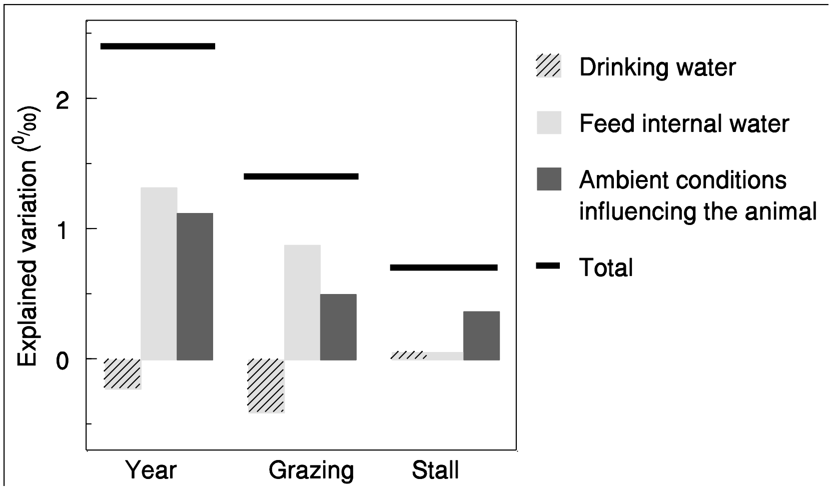

Fig. S3: Explained variation of δ_{hair} by different parameters in whole years, grazing and stall seasons (the parameters contributing little to the variation are not shown here). Lines represent the total variation of $\delta^{18}O$ in hair in whole years, grazing and stall seasons. Note that the effect of ambient conditions influencing the animal does not include the effects on plants. The sum of feed and ambient conditions is larger than total variation because drinking water intake compensates some of both effects.

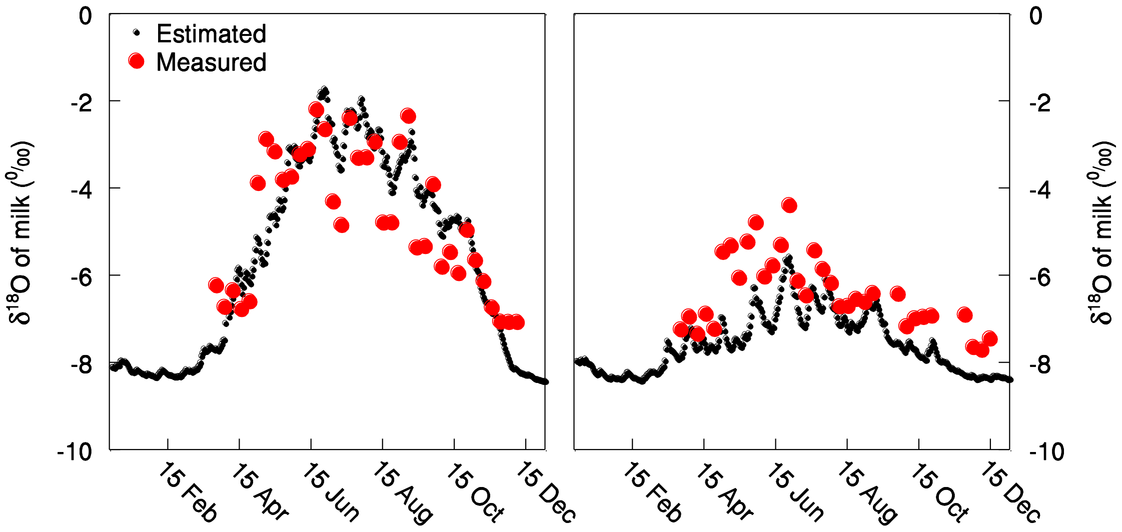

Fig. S4: Measured and predicted $\delta^{18}O$ in milk water during a year for two farms differing considerably in milk yield: Left farm: 14 kg d^{-1} annual average per cow; right farm: 26 kg d^{-1} annual average per cow. Note: the large difference between both farms is not caused by the difference in milk yield, which has a marginal influence, but it is caused by the exclusive provision of fresh grass in the left farm during the growing season while the right farm provides no fresh grass but constant feed (total mixed ration) throughout the year.

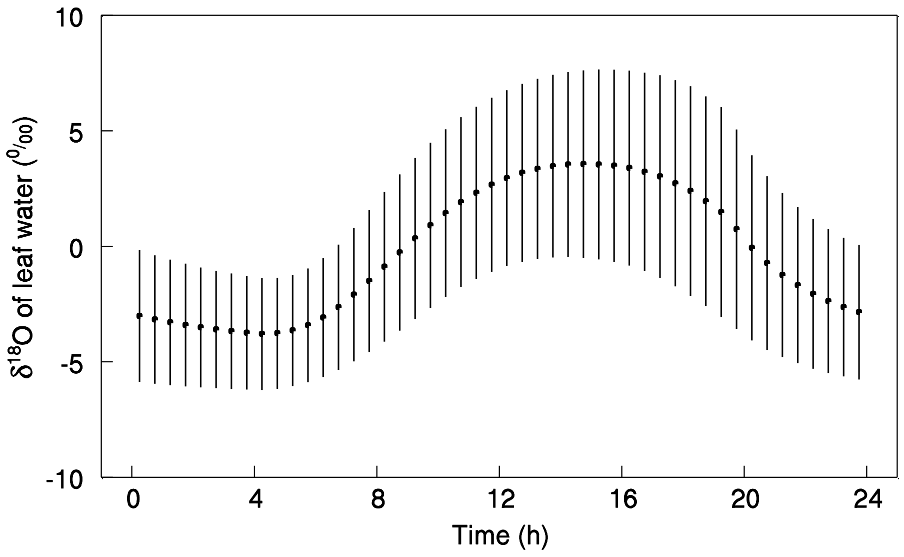

Fig. S5: Diurnal of $\delta^{18}O$ in leaf water estimated by MuSICA. Note: points and lines denote average values and standard deviations during five grazing seasons.

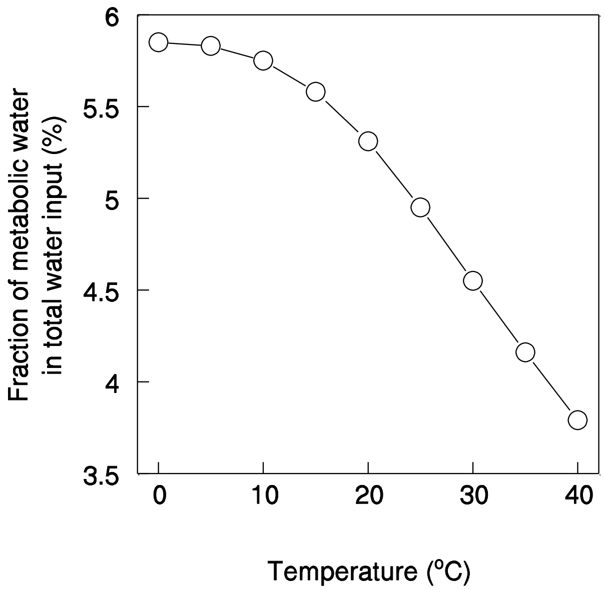

Fig. S6: Influence of temperature on the fraction of metabolic water as predicted by the MK model under otherwise constant conditions.
